# Innovations in cortical and subcortical stimulation mapping

**DOI:** 10.3171/2024.10.FOCVID24144

**Published:** 2025-01-01

**Authors:** Richard W. Byrne, Isabelle Germano, Joao P. Almeida, Shawn Hervey-Jumper, Emmanuel Mandonnet

**Affiliations:** 1 Department of Neurosurgery, Mayo Clinic, Jacksonville, Florida;; 2 Department of Neurosurgery, Icahn School of Medicine at Mount Sinai, New York, New York;; 3 Department of Neurological Surgery, University of California, San Francisco, California;; 4 Department of Neurosurgery, Lariboisière Hospital, Paris, France; and; 5 Department of Neurosurgery, Indiana University, Indianapolis, Indiana

Surgical interventions in eloquent areas of the brain have become more commonplace as surgeons use extraoperative and intraoperative technologies to improve safety. Chief among these strategies remains the technique of cortical and subcortical stimulation mapping. In this edition of *Neurosurgical Focus: Video*, selected innovative videos are presented, allowing our readers to evaluate the latest in stimulation mapping innovation. Dynamic mapping techniques are best demonstrated using a dynamic format such as video.

Six unique videos were chosen, and each has a different emphasis. The various works allow the viewer to learn different aspects of cortical stimulation mapping. In a classical video report, Yindeedej et al. demonstrate their specific protocol for cortical stimulation mapping of a low-grade glioma in the dominant-hemisphere temporoparietal junction. Avoiding new deficits during the resection is critical in such cases. Kappel et al. demonstrate their technique for mapping during high-grade arteriovenous malformation surgery. This is an underutilized approach in most institutions treating arteriovenous malformation. Their technique is well depicted in the video. Vellore et al. describe their "hexamodal" mapping technique. Their dominant-hemisphere cortical malformation epilepsy case required mapping of motor, sensory function, speech, visual function, cognitive function, and epilepsy outcomes. Hamer et al. demonstrate simultaneous cortical and subcortical stimulation mapping with arcuate fasciculus cortico-cortical evoked potentials. More examples of this technique will be needed to prove its efficacy. Gecici et al. show their elaborate methods for mapping neuropsychological function during awake surgery. This is an area of exploration at many centers, with considerable variability in technique and outcomes. Finally, in what is likely the most innovative video brain mapping case, Guo et al. demonstrate the potential value of performing awake posterior fossa motor mapping for a residual low-grade glioma invading the brainstem. The patient did well and tolerated the procedure without difficulty. This technique is rarely used in posterior fossa surgery, but with modern anesthetic techniques, the approach is safer now than it was in the past. It is likely that stimulation mapping in the posterior fossa for intra-axial lesions adjacent to the pons is an underutilized technique. This approach can also be done under general anesthesia, but the authors performed the procedure with the patient awake for more reliable feedback.

We hope you find these state-of-the-art videos as interesting to watch as we did when reviewing them. You may consider adopting some of these techniques in your clinical practice.

## Disclosures

Dr. Germano reported consulting for Brainlab outside the submitted work.

