# Introduction. Innovations in cortical and subcortical stimulation mapping

**DOI:** 10.3171/2025.2.FOCVID24144a

**Published:** 2025-04-01

**Authors:** Richard W. Byrne

**Affiliations:** Mayo Clinic, Jacksonville, FL

TO THE READERSHIP: An error appeared in the article by Byrne et al. (Byrne RW, Germano I, Almeida JP, et al. Introduction. Innovations in cortical and subcortical stimulation mapping. *Neurosurg Focus Video*. 2025;​12[1]:V1).

It was incorrectly stated that the case by Guo et al. was performed while the patient was awake; this case was performed under general anesthesia. The corrected sentence appears below.

Finally, in what is likely the most innovative video brain mapping case, Guo et al. demonstrate the potential value of performing posterior fossa motor mapping under general anesthesia for a residual low-grade glioma invading the brainstem.

The article has been corrected online as of April 1, 2025.

